# Elective courses for medical students during the preclinical curriculum: a systematic review and evaluation

**DOI:** 10.3402/meo.v20.26615

**Published:** 2015-05-11

**Authors:** Ankit Agarwal, Stephanie Wong, Suzanne Sarfaty, Anand Devaiah, Ariel E. Hirsch

**Affiliations:** 1 Department of Radiation Oncology, Boston University School of Medicine, Boston, MA, USA; 2 Office of Academic Affairs, Boston University School of Medicine, Boston, MA, USA; 3 Department of Otolaryngology, Boston University School of Medicine, Boston, MA, USA

**Keywords:** humanities, education, medical student, electives, undergraduate, preclinical, curriculum

## Abstract

**Objective:**

Preclinical medical student electives are prevalent at medical schools across the United States, but the range of electives available and their impact on medical student education are not well described in the literature. The objective of this article is to review the literature relating to preclinical medical student electives and their impact on medical student educational outcomes.

**Methods:**

We reviewed studies that met the following criteria: English-language articles describing preclinical US-based medical electives. We used PubMed journal databases and limited our search for the time period 1999–2014. We excluded electives based in other countries or electives designed for third or fourth year students. Data abstracted included the topic of the elective, qualitative descriptions of the electives, and any associated surveys or exam data associated with the electives. Data were synthesized using descriptive tables sorting electives by broad topic. Reported outcomes and statistical methods were analyzed to assess study quality.

**Results:**

We found a wide range of subjects taught in the form of preclinical medical school electives. We identified electives in clinical skills, the humanities, student lifestyle, specialty-specific electives, and an assortment of other miscellaneous electives. Surveys and exams administered to students showed that the electives were universally well received by students. Of the 37 electives identified, 15 electives used quantitative objective assessments, such as knowledge exams, while the remaining tended to use student self-reported results.

**Conclusions:**

Preclinical medical student electives are prevalent at medical schools across the United States and have a significant impact on medical student education.

Since the publication of the Flexner Report in 1910 and the relative standardization of medical education across the United States, most medical schools have offered a standard 4-year curriculum. This curriculum typically includes training in the basic sciences for the first 2 years followed by clinical rotations in the final 2 years. Recently, medical schools have been implementing alternative curriculum pathways. These include shortened preclinical curricula, dedicated research time during medical school, and early clinical exposure experiences. Although a strong foundation in basic science and clinical medicine remains essential, medical schools have increasingly dedicated educational time to behavioral and social sciences due to a recognized need for more training in those domains (1–3). Given the constraints of an expanding body of knowledge, varied medical student interests, and a limited amount of educational time, many medical schools now offer elective courses that students can pursue based on their personal interests.

Electives can contribute to both the professional and personal development of medical students in specific areas of interest outside of the standard curriculum (1, 4, 5). Medical schools across the United States offer electives in areas such as leadership, ethics, health policy, business, foreign languages, literature in medicine, and specialty-specific electives. Whereas some medical schools only offer electives led by faculty members, many medical schools also include electives primarily organized and run by medical students, including our own institution.

Reviews currently found in the literature are limited, often focusing on a subset of electives such as international health experiences and/or senior year clinical electives (6, 7). This paper provides a systematic review of US-based preclinical medical electives described in the literature between 1999 and 2014. The aim of this systematic review is to 1) describe the range and content of preclinical electives as reported in the current literature, 2) report medical student assessments of these electives, and 3) establish and compare the impact of different categories of preclinical electives on improving knowledge, skills, or behaviors in preclinical medical students who participated in these electives compared to their peers who did not.

## Methods

### Search strategy

A systematic literature review was conducted to identify English-language articles describing preclinical US-based medical electives, using PubMed journal databases. Our team consisted of medical students and faculty of different specialties and members of the Office of Academic Affairs at our institution. We collaboratively defined ‘preclinical electives’ as optional, pre-clerkship supplemental academic experiences that students could choose to sign up for during their first and second year, as opposed to experiences that were during the clerkship years or required portions of the academic curriculum. To identify these electives, search terms including ‘medical electives’, ‘medical track’, ‘medical pathway’, ‘preclinical [or] first year [or] second year’, ‘medical students’, as well as specialty-specific terms like ‘surgery’, ‘primary care’, ‘family medicine’, ‘emergency medicine’, ‘radiology’ were used. After piloting the review with these search terms, we limited our results to the last 15 years, for the period of 1999–2014, in order to collect an accurate reflection of the current status of electives in medical school curricula.

### Inclusion and exclusion criteria

We only included articles that described electives that had didactic material and were offered during first or second year of US medical schools. We excluded electives based in other countries, electives offered exclusively during the summer, electives that offered purely experiential experiences (such as community service or global health programs) without any associated didactic teaching or discussion sessions, and electives designed for third or fourth year medical students and/or non-medical students.

### Data extraction and synthesis

Two reviewers initially independently performed the search. One reviewer then performed an initial analysis of each article, extracting and summarizing the structure, content, and outcomes of each elective. A second reviewer duplicated the review of each article to ensure consistency in reporting, and further categorized and organized the results. Ultimately, results were stratified by type using the categories specialty specific, student skills, humanities, student lifestyle, reproductive health, and other. We used part of the Medical Education Research Study Quality Instrument (MERSQI) criteria when describing the outcomes of each elective, based on type of data and what was measured (8). The initial review was conducted by reviewers with no direct experience with any of the published electives to minimize sources of bias.

## Results

We identified articles that described 37 preclinical electives for medical students between 1999 and 2014. Of these articles, we identified 20 specialty-specific electives, three student skills electives, five humanities electives, four lifestyle electives, two reproductive health electives, and 11 other miscellaneous electives. Since there are no previous comprehensive reviews of preclinical medical electives, we arrived at these classifications collaboratively with our co-authors. Our goal was to create categories that were distinct enough so that our results and discussion could focus on characteristics unique to that category, while keeping the subsets large enough to be manageable from the perspective of the reviewers as well as readers.

We used part of the MERSQI score when evaluating outcomes of each elective. Specifically, we describe what was measured (i.e., satisfaction, perception, general facts vs. knowledge, skills vs. behaviors) and how outcomes were assessed (self-assessments vs. outcomes).

### Specialty specific

We identified several articles describing electives designed to expose students to specific specialties (Supplementary Tables 1–4). Specifically, we identified two articles describing electives in emergency medicine (9, 10), four articles describing primary care electives (11–14), two articles describing radiology electives (15, 16), and six articles describing four surgery electives (17–22).

#### Structure and content

Specialty-specific electives utilized lectures by faculty, skills session, clinical observation time, and case-based discussions. The emergency medicine electives typically included clinical time in the ambulance and/or emergency department in addition to teaching sessions focusing on critical care medicine and common procedures (9). The primary care electives generally had a stated goal of increasing the number of students interested in entering primary care. The structure of the electives varied widely but included clinical exposure to primary care, clinical skills workshops, lectures by primary care preceptors, and career advice sessions. Some of the lecture topics included introduction to clinical skills, patient education, health care teams, health maintenance, ethics, cultural determinants of health care, and food/nutrition (11). The radiology electives used case-based discussions and introduced students to the proper uses, and associated risks, of using specific imaging modalities in clinically relevant situations (15, 16). The surgery electives included lectures, clinical skills sessions, and operating room experience. Students practiced their operative skills on anesthetized pigs, human cadavers, and in live operating room experiences (17–22).

#### Outcomes

Nine out of the 12 electives used subjective reports from the participants to assess student perception of the electives and/or the specialty as a career, and overall were quite positive. A common goal among many of the specialty-specific electives was to increase student interest in the specific specialties. Indeed, several of the emergency medicine, primary care, and surgery electives reported either increased student interest to enter the fields and/or actual matching into those fields following completion of the electives (9, 11–13, 20, 21–23) . In addition, the electives that focused on clinical skills sessions and live clinical experiences universally demonstrated increases in students’ self-reported confidence in their clinical skills (9, 13–15).

There were several electives that also used objective measures to assess student knowledge and skills, all of which reported quantitative improvement. Four used objective assessments and/or postcourse examinations (10, 15, 20, 21, 24). Two used subsequent grades in other courses, comparing results to non-participants, to assess knowledge or skill differences (10, 19). Five also looked at how many students matched into the specialty of the elective, though only the three primary care electives found a significant increase in the number of students matching into the field of interest.

### Student skills

We identified three student skills electives which we defined as electives designed to teach students general skills not specific to any one specialty but to help students during clinical clerkship rotations (24–26) (Supplementary Table 5).

#### Structure and content

The skill sets taught by the three electives varied. One elective focused on the traditional skills of the history and physical exam. It also provided clinical overviews of common clinically focused topics such as hypertension, diabetes, myocardial infarction, breast cancer, and coma. The course included didactic lectures, small group discussion sections, and clinical time in the hospital to allow students to practice their physical exam skills. Another elective was designed to improve students’ interview skills through improvisation. The sessions included improvisational exercises, large-group reflective discussion, and small group projects. Topics covered included listening, affirmation, vocal tone modulation, nonverbal communication, agreement, collaboration, acceptance, and validation. The third elective was designed to improve students’ research skills particularly as it pertains to clinical clerkships. Students learned about the different point-of-care research resources available to them and how to effectively use these resources to answer clinical questions.

#### Outcomes

All three of the electives used subjective surveys following the course to assess student impressions. All three were well received by students and saw increases in student self-reported confidence in using the skills taught in the clinical care of their patients. Morley et al. used pre- and postassessments to demonstrate improved skill level, particularly in the use of RefWorks and website evaluation (24).

### Humanities

We identified five articles describing electives in the humanities (Supplementary Tables 6 and 7). Two electives focused on end-of-life care (27, 28). Three of the humanities electives focused on creative writing & literature (29–31).

#### Structure and content

Students in both end-of-life care electives were paired with seriously ill patients or patients from local hospices that students would regularly interact with throughout the semester. Both electives incorporated regular follow-up reflection sessions through small group discussions. One of the electives additionally included large-group lecture or exercise sessions covering specific topics such as The Dying Experience, Breaking Bad News, Spirituality in End-of-Life care, Childhood Death and Dying, Advanced Directives, Hospice and Palliative Care Medicine, and Bereavement.

The creative writing and literature electives focused on medically oriented literature to explore themes such as memorable patient encounters, professionalism (including empathy and compassion), pain, sexuality, and the doctor–patient relationship. One of these electives focused on understanding the different points of view in the texts, including those of physicians, patients, and the family members (31).

#### Outcomes

Self-assessments were used to evaluate outcomes for all of these electives. The end-of-life-care elective that had reported quantifiable outcomes found that participants felt less concern about working with dying patients and increased their assessment of clinical criteria when thinking about a ‘good death’ compared to non-participants (28). The electives in creative writing and literature demonstrated increases in student empathy and student understanding of the patient's perspective on medical care (30, 31).

### Student lifestyle

We identified four articles describing student lifestyle electives which we defined as electives designed to improve medical student lifestyles, often through stress reduction and meditation (32–35) (Supplementary Table 8).

#### Structure and content

The primary goal of these electives was to reduce stress in medical students. One elective approached this through a series of 1-hour lectures providing information on wellness, stress reduction, and coping strategies. Students wrote a two-page postcourse self-reflection essay describing their own stresses, and discussing their present coping behaviors. In the second elective, 30-min didactic sessions were followed by small group activities relating to the day's topic such as ‘eating mediation’, drawing self-portraits, and reflecting on current stressors. Didactic sessions covered theoretical concepts behind the stress response, meditation, imagery, exercise, nutrition, genograms, and spirituality. Furthermore, students were assigned ‘homework’ consisting of physical activity lasting at least 30 min, three times a week and meditation for 15 min, 6 days a week. The third elective similarly included didactic based sessions and group discussions on topics including biofeedback, imagery, meditation, and others. The fourth elective was built around hour-long yoga sessions during which students learned breathing and meditation exercises. This was followed by a 30-min lecture about the neuroscience of yoga, relaxation, and breathing exercises.

#### Outcomes

These wellness electives encouraged students to engage in personal wellness activities. Three out of four electives assessed outcomes with self-assessments. One elective demonstrated quantitative reductions in anxiety among participants. Furthermore, these decreased anxiety levels were sustained for 3 months following the conclusion of the course (32). The yoga elective demonstrated statistically significant increases in self-regulation and self-compassion, and suggestions of improvements in empathy and perceived stress, although those did not reach statistical significance (33).

Uniquely, Maclaughlin et al. assessed outcomes through quantitative biochemical analyses of stress hormone samples. Cortisol, dehydroepiandrosterone-sulfate (DHEA-S), testosterone, and secretory immunoglobulin A (sIgA) were measured in saliva samples from participants and non-participants. Stress hormone levels were significantly less in participants compared to non-participants at the end of the intervention.

### Reproductive health

We identified two reproductive health electives, which we defined as electives that covered topics in sexual health, abortion, and other reproductive health topics (36, 37) (Supplementary Tables 9 and 10).

#### Structure and content

Both electives were primarily lecture based. One elective focused primarily on abortion education and sexual health, consisting of 10 lectures with guest speakers. Students were also required to attend a half-day shadowing experience at a local abortion clinic. The other elective was a longer 9-week course focused on teaching core competencies in reproductive health, several of which correlated with competencies identified by the Association of Professors of Gynecology and Obstetrics (APGO). Students were also required to present topics at a reproductive health fair at the end of the course.

#### Outcomes

Both electives were well received by students. Both electives used self-reported surveys to assess outcomes. Caro-Bruce et al. used five-point Likert scales (1=excellent, 5=inadequate/poor) to specifically assess students’ perception of lecture quality, and found that the course overall scored 1.8, and scored 1.9 for the ‘elective enhanced my understanding of the topics’. Meites et al. used yes/no postcourse surveys and found that the majority of students felt increases in comfort discussing reproductive issues such as contraception and sexual orientation.

### Other subject specific electives

We identified 11 other miscellaneous subject specific electives (Supplementary Table 10). These varied widely in terms of subject matter and format. Topics included cultural competency training, chronic hepatitis B (a model for health disparity), oral health, air medical transport, a book discussion group, the healers art, health policy and legislation, adolescent maltreatment, refugee health, rural medicine, and quality and safety (36, 38–48).

#### Structure and content

The structure and content of these courses varied widely. Most courses included lectures by faculty physicians and local experts on the topic in addition to some form of clinical exposure related to the subject. Some courses, such as the quality and safety course and the rural medicine courses, required students to create their own projects or presentations related to the subject.

#### Outcomes

Most courses used subjective surveys and/or objective question-based tests to measure student perceptions of the course and increases in knowledge. All of the courses were positively received by students. The three courses that used objective question-based test measures showed overall statistically significant increases in the subject matter knowledge (40, 41, 44).

### Student-initiated electives

Several of the electives discussed were student-initiated, ranging from reproductive health to student skills; however, there were no student-initiated procedural-based electives. Two of the electives, in end-of-life care and student skills, specifically identified students as actively involved in teacher roles (25, 27). Participants in the student skills elective specifically stated that they appreciated having a peer as the facilitator. All of these electives reported increased interest and/or proficiency in their respective subjects.

### Overall outcomes

All the studies reviewed reported that the preclinical electives were positively received by students. Of the 37 electives identified, a total of 27 studies employed surveys to assess student impressions of the elective (13–22, 24–32, 36, 37, 39–42, 44, 45, 47–49) . Fifteen of the electives used quantitative objective assessments, such as knowledge exams, residency placement results, and validated quantitative scoring rubrics (11, 12, 15, 17, 18, 24, 31–33, 35, 40, 41, 44, 45).

## Discussion

This paper attempts to update the current literature on preclinical medical student electives by presenting a systematic review of electives described in the literature since 1999. Our review highlights the wide range of preclinical electives available at institutions across the United States. We also achieved our aim of evaluating outcomes of these different electives. The electives increase medical student knowledge in areas outside of the traditional medical school curriculum, teach medical students useful skills, increase student wellness, and impact eventual career choices. Each elective that we reviewed employed the use of student data and other measurable outcome measures in order to inform readers and implement internal changes to the electives. However, we did find great variation in the type and quality of outcome assessment measures reported by each article. The MERSQI score assigns a greater value to outcome measures of behavior, knowledge, and skills compared to satisfaction and perception as a reflection of quality in medical education research (8). Yet, we found that most electives used self-reports to assess student perception about the elective content, a minority used objective measurements to quantitatively demonstrate the acquisition of knowledge or skills, and fewer still demonstrated changes in subsequent behavior (i.e., match results). While student self-surveys of these electives have shown that the electives are well received by students, we recognize this could be a matter of self-selection among students who might already be enthusiastic about the elective content. To address the issue of self-selection and create a control group, future researchers can administer assessments to students who took the elective as well as those students who chose not to. In addition, researchers can focus on objective assessments measuring the effect of the elective on knowledge and skills, or outcomes data assessing the impact of the elective on future behaviors such as career choice.

### The process of implementing an elective

Most elective courses are developed through the work of medical students and faculty who are passionate about topics not typically covered in the standard medical school curriculum. The first step in creating a successful elective is to determine whether there is student interest in the topic. Student interest can be gauged through formal surveys to the student body using electronic survey software. These surveys are useful in assessing a baseline knowledge level, which can be used to establish course content. The next step is to assess the ideal course structure. This paper has highlighted a multitude of ways to structure a course through the electives reviewed. Additionally, we have found that course directors from outside institutions are typically open to sharing the course syllabi when contacted. The concurrent step is to develop a curriculum and compile the course materials. Generally, successful courses maximize the use of elective time without requiring extensive amounts of outside work for the class, in deference to students’ workload. However, courses such as medical foreign language courses may require more self-study than others. For courses that utilize guest speakers, identifying a list of speakers early on is integral to success. Course directors should identify topics and accordingly identify speakers from both within and outside of the medical school faculty. Similarly, for electives that are more clinically based, course directors should identify preceptors and sites early on that are appropriately in line with the elective topic. Once the course is organized, course directors can advertise through elective course listings on the school website, through lunch events before the course begins, and through emails to the medical student email lists. Finally, course directors can benefit from employing student surveys at the end of the course to help identify ways to improve the course for future years.

### Measuring outcomes of an elective

The MERSQI paradigm for medical education research provides an informative guide to conducting quality medical education research. The MERSQI emphasizes the use of knowledge assessments in a semi-controlled way to gauge the impact of an educational intervention. The majority of electives we reviewed assessed the impact of the elective through either a knowledge exam or a student perceptions survey.

The knowledge exam consists of an assessment, which can be administered to participants before and after the course. The exam should include questions that directly test the course's learning objectives. To reduce bias, the exam can include control questions that are not addressed in the coursework, and the exam can be administered to students who did not participate in the elective course. The knowledge exams can help the course directors determine how effective the course is in teaching to the learning objectives and help course directors address deficiencies in the curriculum in subsequent years.

Most student surveys used a Likert scale to assess how students feel about specific lectures, how well prepared students feel after completion of the elective, and how students feel about the elective overall. Positive survey results can be used to advertise the course to future students and negative results can be used to make changes to the course in future years.

Measuring outcomes is helpful in facilitating internal review of the elective. However, beyond internal review, measuring outcomes is also useful to justify the course the school administration, satisfy outside funding sources, and publish results.

## Conclusion

There is a wide variety of preclinical elective courses described in the literature. These electives play an important role in medical students’ education by increasing medical student knowledge in areas outside of the traditional curriculum, teaching medical students useful skills, increasing student wellness, and impacting eventual career choices. While this does not necessarily mean that institutions should substitute formal curricular content for electives, electives do have a measurable, positive impact and can be a useful complement to the formal curriculum. Support for faculty and students interested in implementing preclinical electives should be a priority for institutions for medical schools interested in training well-rounded physicians.

To this end, by highlighting the diversity in structure and breadth of subjects potentially available to preclinical medical students, the contribution of this work is to provide a resource and stimulus for the development of other electives at individual institutions. We also hope that this work will encourage improvements in the quality of future published reports of preclinical electives.

## Supplementary Material

Elective courses for medical students during the preclinical curriculum: a systematic review and evaluation
