# Supplementary material for: Elective courses for medical students during the preclinical curriculum: a systematic review and evaluation
Source: Med Educ Online. 2015 May 11;20:10.3402/meo.v20.26615. doi: 10.3402/meo.v20.26615 (PMC4429260; doi:10.3402/meo.v20.26615)
Supplement: Elective courses for medical students during the preclinical curriculum: a systematic review and evaluation [file MEO-20-26615-s001.pdf]

SUPPLEMENTARY MATERIAL

Supplemental Table 1: Medical Student Electives in Emergency Medicine

**Study**	**Structure of Course**	**Outcomes of Course**
Chapman,1999 Impact of EMS education on emergency medicine ability and career choices of medical students	▪ Spanned four months (one semester), and included classroom and laboratory presentations for two hours per week, three evening ride-along shifts, and one night in the emergency department.<br>▪ Was designed to introduce students to pre-hospital emergency care, specifically Emergency Medical Service (EMS) content. Students also gained specific skills and knowledge in scene safety, BLS, airway management, cervical spine precautions, vital signs, splinting, bandaging, and suturing through their ride-alongs and ED observations.<br>▪ During the two-hour classroom session, students discussed their pre-hospital experiences with Emergency Medicine residents and staff.	▪ Students took a pre-course and post-course survey and results were analyzed.<br>▪ The total number of students who were generally interested or very interested in acute care-emergency medicine did not change. However, there was a statistically significant increase in students who were interested in Emergency Medicine as a career.<br>▪ Students also reported increased self-perceived competence relating to emergency scenarios.
Van der Vlugt, 2002 Teaching Procedural Skills to Medical Students: One Institution’s Experience With an Emergency Procedures Course	▪ 12 weeks long (one quarter) consisting of 40 classroom hours of lecture model practice, 24 hours of clinical shift in the emergency department, and 8 hours of fresh frozen cadaver lab.<br>▪ Students met for two hours twice weekly. Each class session focused specifically on one or several procedures such as airway management techniques, suturing, thoracentesis, or venipuncture (there were approx. 20 topics total).<br>▪ The first half of each session consisted of lecture or discussion and demonstration. The second half was then dedicated for students to practice the procedure with guidance on mannequins, each other, substitutes, or cadavers.	▪ Students completed a final examination with written and practical components. Furthermore, numeric scores in procedural and medical knowledge categories were extracted from evaluations from internal medicine, surgery, obstetrics and gynecology, and emergency medicine rotations. Participants’ scores were compared with nonparticipants. Participants achieved higher competence scores in procedural skills in all rotations (with statistical significance in Emergency Medicine and Internal Medicine) and increased medical knowledge in most rotations except one month of

Study	Structure of Course	Outcomes of Course
		surgery and OB/Gyn.<br>▪ There was no reported analysis looking at student interest in Emergency Medicine. However, it was noted that there were more students who enrolled in the elective than matched in emergency medicine.

Supplemental Table 2: Medical Student Electives in Primary Care

Study	Structure of Couse	Outcomes of Course
Grayson, 2000 Impact of a First-year Primary Care Experience on Residency Choice	▪ Ten three-hour observation sessions, seven half-day lectures and small group sessions, and readings and required tasks to perform in a preceptor's office.<br>▪ Topics included: introduction to clinical skills, patient education, health care teams, health maintenance, ethics, cultural determinants of health care, food/nutrition<br>▪ Preceptors were from Internal medicine, pediatrics, family medicine, and medicine-pediatrics.	▪ Participants were significantly more likely to enter Primary Care residencies. Specifically, the odds of entering a primary care residency were 40-50% higher among participants vs. non-participants.
Elder, 2006 The art of observation: impact of a family medicine and art museum partnership on student education.	▪ The course used art to hone medical student skills in the art of observation in the context of family medicine<br>▪ Course consisted of several short lectures, group discussions, observation sessions at the local art museum, and clinical sessions shadowing faculty in their family medicine practices	▪ Students were asked to complete open-ended questions via an online survey following completion of the course<br>▪ Students self-reported that the elective increased their awareness of physician biases and improved their nonverbal communication skills
Wilkinson, 2010 FaMeS: An Innovative Pipeline Program to Foster Student Interest in Family Medicine	▪ The FaMeS track has curricular, extracurricular, summer, and career planning aspects.<br>▪ Curricular components included preferential assignment of a family medicine faculty member or site for students' Introduction to clinical medicine (ICM) course and Integrated Problems (IP) course – requirements of the medical school curriculum.	▪ Number of students matching into Family Medicine was compared from 3 years before the introduction of the track to 3 years after the introduction of the track.<br>▪ There was a significant increase in the number of students going into family

Study	Structure of Couse	Outcomes of Course
	▪ Students also received preferential lottery into an optional summer externship in family medicine, which consisted of 3-4 weeks in a family physician's office with online discussions.<br>▪ Extracurricular components included monthly workshops offered throughout first and second year on topics such as the acquisition of clinical skills, or discussions about family medicine as a career. There are also monthly brown-bag lunches to practice Spanish language skills.<br>▪ Students were also encouraged to attend national conferences (AAFP, STFM) with a small amount of funding to reimburse expenses.	medicine. Students after the introduction of the program were approx. 1.94 times more likely to match into family medicine before the introduction of the program.
Keating, 2013 How we created a peer-designed specialty-specific selective for medical student career exploration	▪ Was one of several "selectives" students could pick to attend. It consisted of one week of workshops, faculty and resident panel discussions, and clinical shadowing experiences based on a student needs assessment.<br>▪ Was designed to provide medical students with early exposure to pediatrics. Topics were based on a needs-assessment prior and included an introduction to pediatrics, adolescent medicine, international child health, and community service or research opportunities.<br>▪ Students completed at least three clinical shadowing experiences providing them with firsthand experience practicing in general pediatrics and subspecialty fields of their choice.<br>▪ Some renditions of this elective added child psychiatry interviews, student-led recreational activities for pediatric patients through child life, and parents' panel speaking on the challenges of having a child with a severe medical illness.<br>▪ Students also received hands-on training	▪ Outcomes were assessed by a student survey. In general this elective received positive feedback.<br>▪ Participants reported a heightened interest and expanded knowledge about pediatrics.<br>▪ An average of 8.6 out of 10 students responded favorably to the statement "I am more likely to go into pediatrics as a result of this selective".

Study	Structure of Couse	Outcomes of Course
	in performing basic neonatal procedures including blue baby resuscitation, meconium delivery care, and umbilical line placement.	

Supplemental Table 3: Medical Student Electives in Radiology

Study	Structure of Course	Outcomes of Course
Erinjeri, 2006 Redefining radiology education for first-year medical students: shifting from a passive to an active case-based approach.	▪ The course was taught over five two-hour sessions<br>▪ The course consisted of lectures, case exploration in the radiology library, and informal discussions regarding cases.<br>Students presented their case in groups to other students and faculty as a final project	▪ A seven-question five-point Likert scale survey was administered at the mid-point and at the conclusion of the course<br>▪ Students self-reported a better understanding of the role of the radiologist in clinical care<br>▪ Student interest in radiology as a specialty choice was unchanged but trended towards increased interest
Leschied, 2013 Emergency Radiology Elective Improves Second-Year Medical Students' Perceived Confidence and Knowledge of Appropriate Imaging Utilization	▪ Was one of several “mini-electives” that students could choose from. It was three days long, consisting of three 2-hr sessions.<br>▪ The goal was to teach students the correct imaging test to order for common acute clinical scenarios.<br>▪ The first session covered topics such as introduction to evidence-based imaging, consequences of inappropriate utilization of medical imaging, and radiation risks associated with imaging modalities.<br>▪ The second session consisted of interactive case-based discussions of common emergency department clinical complaints.<br>▪ The final session was dedicated to 5-minute presentations students were required to present on the appropriate imaging algorithm for a clinical emergency scenario.	▪ Students were given pre- and post-course knowledge assessments with case-based MCQ, and included questions on the course itself.<br>▪ Students reported increased self-perceived confidence in ability to select the most appropriate imaging modality to investigate common emergency complaints.<br>▪ There was no effect in the number of students who answered, "Have you considered radiology as a career". However, students did report an increased desire to spend time in a radiology reading room.<br>▪ Students also suggested improvements to the course such as more basic radiology, including going over in greater detail radiology-anatomy correlations and teaching the

Study	Structure of Course	Outcomes of Course
Erinjeri, 2006 Redefining radiology education for first-year medical students: shifting from a passive to an active case-based approach.	▪ The course was taught over five two-hour sessions<br>▪ The course consisted of lectures, case exploration in the radiology library, and informal discussions regarding cases.<br>Students presented their case in groups to other students and faculty as a final project	▪ A seven-question five-point Likert scale survey was administered at the mid-point and at the conclusion of the course<br>▪ Students self-reported a better understanding of the role of the radiologist in clinical care<br>▪ Student interest in radiology as a specialty choice was unchanged but trended towards increased interest
		basics of the utility of various imaging modalities.

Supplemental Table 4: Medical Student Electives in Surgery

Study	Structure of Course	Outcomes of Course
Riboh, 2007 Innovative introduction to surgery in the preclinical years	▪ An eight week elective consisting of hour-long sessions twice per week for a total of 16 sessions.<br>▪ Provided an overview of surgery as a field including range of conditions treated, lifestyle, and training.<br>▪ Students also worked through classic cases of various subspecialties. Over the next few sessions, students learned basic surgical skills and OR procedures. They also performed bowel resection, suture and staple anastomoses, and fascial closure on live anesthetized pigs.<br>▪ Students also had the opportunity to interact with surgery faculty and residents.	▪ Each session was evaluated by anonymous survey. The course was generally well received.<br>▪ Students reported increased confidence and comfort entering surgical clerkship.<br>▪ There was also an overall increase in participation in Surgery Interest Group events, shadowing and research.
Sammann, 2007 A Surgical Skills Elective to Expose	▪ The first part of this elective – called "Operating Room Assist" – was designed to be a surgical skills training and consisted of three 2-hour	▪ Outcomes were assessed with multiple questionnaires.<br>▪ Students reported significant improvement in confidence in

Study	Structure of Course	Outcomes of Course
Preclinical Medical Students to Surgery<br><br>Cloyd, 2008 Operating Room Assist: Surgical Mentorship and Operating Room Experience for Preclerkship Medical Students	sessions (six hours total). The elective included instruction in surgical skills and an overview of OR etiquette. Surgical skills covered included knot tying, surgical instruments, suturing, proper OR technique, laparoscopic camera, and cautery.<br>During the clinical sequel - called “Operating Room Assist: Clinical Experience” – first-year students could choose to be paired with surgical faculty members who invited them into the operating room to assist with various procedures in order to practice the surgical skills the students just learned. Students were required to participate in at least 2 surgeries with their surgeon over a 3-month elective period[3]<br>▪ .	suturing, knots, and instrumentation.<br>▪ Students had less concerns about having joy and enthusiasm in surgery. However, the elective did not significantly change their concerns about the environment or lifestyle in surgery.<br>▪ Likelihood of choosing a career in surgery or subspecialty also did not change significantly.<br>▪ Most students (73.1%) directly participated during their first surgical case. Surgeons rated participants slightly better than third-year clerkship students, and nurses reported that participants needed less instruction and were more familiar with OR practices.
Haubert, 2009 Surgical Clinical Correlates in Anatomy: Design and implementation of a first-year medical school program[18]	▪ A 10-week elective that correlated with the first year anatomy course. It consisted of eight 2-hour sessions including general surgical knowledge, orthopedic surgery, plastic surgery, urology, cardiothoracic surgery, general surgery, vascular surgery, and ENT sessions.<br>▪ Each session included a review of relevant anatomy, clinical correlations, pertinent radiologic studies, and a surgical video. Then, attending surgeons in the specific field were asked to demonstrate procedures pertinent to their specialty on cadavers.<br>▪ Students participated in and performed the procedures on the cadavers for hands-on experience.	▪ Pre-evaluation and post-evaluation questionnaires were distributed to the entire class. The elective was well perceived.<br>▪ Participants had the highest average for all combined anatomy examinations compared to non-participants.<br>▪ Participants also had significantly more positive opinions of surgeons post-course.
Lee, 2009	▪ An eight-week long elective	▪ Pre-and post-elective

Study	Structure of Course	Outcomes of Course
The Utility of Endovascular Simulation to Improve Technical Performance and Stimulate Continued Interest of Preclinical Medical Students in Vascular Surgery<br><br>Lee, 2010 Long-term impact of a preclinical endovascular skills course on medical student career choices	consisting of didactic teaching in the form of reading material and weekly 30-minute lectures covering basic catheter-based interventions, aortoiliac disease, superficial femoral artery disease, renal artery disease, and cerebrovascular disease.<br>▪ Instruction was also provided on arteriography, guide wire manipulation, catheter exchange, and angioplasty and stenting.<br>▪ Students also received weekly 90-minute mentored simulator sessions and practiced carotid, renal, iliac, and superficial femoral artery interventions.<br>▪ Students were required to complete sessions in which they acted as the primary operator of the procedure with direct instruction.	simulator assessments were used consisting of objective procedural measures reported by the simulator, and subjective performance graded by several expert observers utilizing a structured global assessment scale. Students significantly improved in both measures after the elective.<br>▪ There was also a pre- and post-elective survey, which showed a significant increase in students seriously considering vascular surgery as a career option. More than 95% of students reported increased knowledge and interest in vascular surgery.<br>▪ A 1-year follow -up survey showed that 70% were still considering vascular surgery.<br>▪ A 3-year follow up survey showed that there was a persistent, if slightly decreased interest level in vascular surgery, along with other surgical subspecialties, radiology, and cardiology.<br>▪ Of the students who had matched by the time the follow-up study was published, there was a slight increase in students (25.1% from 2009 to 2011 vs 21.9% from 2006 to 2008) who matched in surgery or surgical subspecialties since this particular skills course has been offered but was not statistically significant (P = .4)

Supplemental Table 5: Medical Student Electives in Student Skills

Study	Structure of Course	Outcomes of Course
Josephson, 2002 A New First-year Course Designed and Taught by a Senior Medical Student	▪ Eight two-hour sessions (16 hours total) which focused on a clinically-focus topic such as hypertension, diabetes, myocardial infarction, breast cancer, and coma.<br>▪ The first hour was lecture and small group based. The content included a clinical overview of each session's topic, focusing on classic signs and symptoms, diagnosis, and principles of treatment. This included introducing students to computer resources to explore these evidence-based medicine topics.<br>▪ Teaching also included clinical questioning from the instructor in a manner similar to that which the students would encounter on the wards during their clinical clerkships.<br>▪ The second hour was spent at the hospital with selected patients for physical exam practice. The topics roughly correlated with student curriculum.<br>▪ Each student was also responsible for a 10min PowerPoint presentation.<br>▪ Was designed and implemented by a 4th year medical student.	▪ Students filled out evaluations after each session and at the end of the course. The course scored very positively in a number of areas including appropriateness of material, teaching style, effectiveness of introducing computer resources to explore evidence-based medicine topics, quality of patient sessions, and an overall evaluation.<br>▪ One of the positive aspects students commented on was the fact that the instructor was of the same age group as the students.<br>▪ Average overall rating for the course of 9.7 based on a ten-point scale.
Morley, 2012 'Information Survival Skills': a medical school elective	▪ Five, 1.5-hour sessions (7.5 hours total) taught by two health sciences library faculty.<br>▪ The elective's goals were: (1) understand the changing nature of scholarly communication and online publishing, (2) identify and use resources and strategies for searching current best evidence, and (3) communicate and organize findings using presentation and reference management software.<br>▪ Each session had hands-on exercises such as clinical scenarios that students could expect to encounter during clerkship. Students learned of	▪ Students were given evaluations to assess outcomes. All students found the course useful, though 3rd year students found this more helpful than 2nd year students. The authors suggested that this was because 2nd year students had no frame of reference of the usefulness of research skills in the context of clinical rotations. Overall, the elective received high marks, especially in content areas and organization.<br>▪ Students were also given pre-

Study	Structure of Course	Outcomes of Course
	different resources and how to determine which were appropriate in order to answer a particular research or clinical questions.<br>▪ Sessions also included group discussion, paired activities, and brief didactic lectures.<br>▪ The elective culminated into a final oral presentation.	and post-course tests. Students improved in many areas. The greatest increase in skill level was seen in the use of RefWorks, followed by website evaluation.
Sochet, 2013<br>'Thinking on my feet': an improvisation course to enhance students' confidence and responsiveness in the medical interview	▪ Four 2-hour weekly sessions, which introduced and allowed students to practice the principles and skills of improvisation, with an eye toward how these skills could apply to communication with patients.<br>▪ Sessions included improvisational exercises, large group reflective discussion, and small group projects.<br>▪ Specific skills that were practiced and emphasized included listening, affirmation, vocal tone modulation, nonverbal communication, agreement, collaboration, acceptance and validation.<br>▪ Students also created their own improvisation exercises targeted at specific communication skills.	▪ Students were given post-course surveys to assess outcomes. Overall the course was received positively.<br>▪ Twenty-two (81%) rated their enjoyment as 'tremendous'. The desire to experience something new and different from the standard medical curriculum served as the motivation for many of the students (67%) to sign up for the course.<br>▪ Most students (23/27; 85%) thought that the concepts that were addressed were either 'very much' or 'tremendously' relevant to the care of patients.

Supplemental Table 6: Medical Student Electives in End-of-Life Care

Study	Structure of Course	Outcomes of Course
Taylor, 2003<br>A Student-Initiated Elective on End-of-Life Care: A Unique Perspective	▪ Set during the Spring of 2nd year, and was built around patient encounters with “lessons taught by hospice patients in the comfort of their own homes” (exact number of sessions is unclear).<br>▪ Students were paired with patients recruited from local hospices and acted as mentors.<br>▪ With each patient encounter, students had preparatory meetings and follow up small group discussions. Small group meetings included creative activities such as	▪ Students completed a self-assessment though specific outcomes were unreported.<br>▪ There was general agreement that this course filled a need for end-of-life care education in their curriculum.

Study	Structure of Course	Outcomes of Course
	role-plays, meditations, and drawing exercises to address issues such as relationship development, emotional support, and spiritual awareness.<br>▪ Upon completion of the course, many participants then graduated to “peer facilitators”, who helped lead the following year’s participants.	
Schwartz, 2005 Detecting attitudinal changes about death and dying as a result of end-of-life care curricula for medical undergraduates	▪ Was called “Caring for the Seriously Ill”, and was taught through seven evening classes spanning across six months.<br>▪ The goals of the course was to (1) familiarize students with principles of end-of-life (EOL) care; (2) increase the comfort level of students in approaching incurably ill and terminal patients; (3) to stimulate students to explore their own attitudes about death.<br>▪ The first hour of each session consisted of an interactive whole-group exercise in the format of a lecture, discussion, or interview by faculty, patients, and/or patients’ family members. Topics consisted of The Dying Experience, Breaking Bad News, Spirituality in End-of-Life care, Childhood Death and Dying, Advance Directives, Hospice and Palliative Care Medicine, and Bereavement.<br>▪ The next hour consisted of preceptor-facilitated small group discussion-based sessions with reflection.<br>▪ Students also were paired with a seriously or terminally ill patient, and would meet periodically throughout the duration of the course.<br>▪ At the end of the course, students were required to write a short paper reflection.	▪ Two self-report measures were used to assess outcomes, called “Concepts of a Good Death” and “Concerns About Dying”.<br>▪ Compared with non-participants, participants reported less concern about working with dying patients and increased their valuation of clinical criteria when thinking about a “good death”.

Supplemental Table 7: Medical Student Electives in Creative Writing and Literature

Study	Course Structure	Course Outcomes
Hatem, 2001 Becoming a doctor: fostering humane caregivers through creative writing	▪ Consisted of seven biweekly sessions each lasting 1.5 hours.<br>▪ Before each session, students wrote narratives or poetry on suggested topics in advance, and then read and reflected on these during the session.<br>▪ Topics included significant experience in medical school, a memorable patient, writing from patient perspective, decision to become a doctor, and different interactions.	▪ The authors identified nine unifying themes when students' papers were analyzed. These themes were: Medicine as a calling, Role confusion and conflict, Identification with the patient, Developing a professional identity, Physician privilege and power, Humanizing the teacher, Death and dying, The limits of Medicine, and Anticipating future challenges.<br>▪ Students also filled out evaluations of the course. Overall, students rated the course favorably. They felt value in the time used to reflect on their experiences, to understand themselves better, and hear other point of views.
Shapiro, 2003 Can Poetry Make Better Doctors? Teaching the Humanities and Arts to Medical Students and Residents at the University of California, Irvine, College of Medicine	▪ A 12-week elective that added optional portions to supplement required a humanities curriculum during first year.<br>▪ Each session consisted of small-group discussion, almost always facilitated by an interdisciplinary team of physician and non-physician.<br>▪ The goals of this elective included enhancing aspects of professionalism (i.e. empathy, altruism, compassion, and caring toward patients), and honing clinical communication and observational skills.<br>▪ Students participated in creative projects to reflect on patients and themselves. Students used prose and poetry to study more in depth patient care issues and other topics covered in the required humanities curriculum.	▪ Standard anonymous evaluations were used with medical students, residents, and faculty to assess the full program as a whole. Overall there was moderate to high level of satisfaction with the program.<br>▪ While results specific to the elective were not available, the authors reported that certain aspects of the humanities medical student curriculum showed significant improvements in self-reported empathy and attitudes regarding the usefulness of humanities to ongoing professional development.

Study	Course Structure	Course Outcomes
Shapiro, 2004<br>Teaching Empathy to First Year Medical Students: Evaluation of an Elective Literature and Medicine Course	▪ Consisted of eight 1-hour sessions twice per month taught by a PhD psychologist and a rotating primary care physician..<br>▪ Students were either assigned to an immediate participation group or a delayed participation group (starting six months later).<br>▪ The elective consisted of on-site readings of poetry, skits, and short stories address the following topics: doctor-patient relationship, physical examination, listening to patients, pain, sexuality, cross-cultural issues, lifestyle modification/noncompliance and geriatrics.<br>▪ During each session, the instructors placed special emphasis on understanding of different points of view in the texts, including those of physicians, patients, and family members, as well as their own.	▪ Quantitative empathy and attitude measures were used to assess outcomes.<br>▪ The splitting of students into immediate and delayed groups controlled for the effects of exposure to standard curriculum and the passage of time.<br>▪ Empathy and attitudes toward the humanities improved significantly after participation in both groups.<br>▪ Student understanding of the patient's perspective became more detailed and complex, as evaluated by qualitative surveys.

Supplemental Table 8: Medical Student Electives in Student Lifestyle

Study	Course Structure	Course Outcomes
Lee, 2001<br>Students' perception of medical school stress and their evaluation of a wellness elective	▪ This Wellness Elective consisted of six 1-hour weekly lectures providing information on wellness, stress reduction, and coping strategies.<br>▪ Students then wrote a two page post-course essay critiquing the elective, describing stresses, and discussing present coping behaviors	▪ Unifying themes from student essays were identified, and included: (1) wellness issues should be important for physicians; (2) their own wellbeing had been diminished by the burden of information to be learned in medical school; (3) talking to peers was a useful coping mechanism, and (4) The elective gave permission to engage in wellness activities without additional guilt.
Finkelstein, 2007<br>Anxiety and stress reduction in medical education: an	▪ Consisted of ten weekly 2-hour sessions, each starting with a 30-min large-group didactic presentation,<br>▪ Didactics covered theoretical concepts behind the stress response, meditation, imagery, exercise,	▪ Several quantitative previously established scales for anxiety, depression, mood, and perceived stress in medical school were used to assess outcomes.

Study	Course Structure	Course Outcomes
intervention	nutrition, genograms and spirituality.<br>▪ This was followed by small group discussion, which consisted of activities related to the day's topic such as "eating meditation", drawing self-portraits reflecting current stressors, etc.<br>▪ Homework expectations included physical activity lasting at least 30 minutes, 3 times a week and meditation for 15 minutes, 6 days a week.	▪ At baseline, participating students had higher initial anxiety scores than students in the comparison group.<br>▪ Anxiety in the study group declined significantly during the course, with enrolled students becoming indistinguishable from non-enrolled counterparts.<br>▪ These decreased anxiety levels were sustained for 3 months following the conclusion of the course.
Maclaughlin, 2011 Stress biomarkers in medical students participating in a mind body medicine skills program.	▪ The course included 11 sessions that included skills-based and experiential learning as well as group discussions.<br>▪ Topics included biofeedback, imagery, meditation, and others	▪ Saliva samples were collected before the course and following completion of the course from both participating and non-participating students<br>▪ Cortisol, dehydroepiandrosterone-sulfate (DHEA-S), testosterone and secretory immunoglobulin A (sIgA) were measured to quantify student stress levels<br>▪ Stress hormone levels were significant less for the intervention group than for the control non-participating student group at the end of the intervention<br>▪
Bond, 2013 Embodied health: the effects of a mind-body course for medical students	▪ Consisted of eleven weekly 1.5 hour sessions during which students learned breathing and meditation exercises, and participated in hour-long yoga sessions.<br>▪ This was followed by a 30 minute lecture about the neuroscience of yoga, relaxation, and breathing exercises.<br>▪ Students received assignments of to practice these techniques at least three times per week.<br>▪ Students also reflected on the	▪ Pre-established quantitative assessment tools were used to evaluate students in four areas: empathy, perceived stress, self-regulation, and self-compassion.<br>▪ There were statistically significant increases in self-regulation and self-compassion, and suggestions of improvements in empathy and perceived stress, though not statistically significant.

Study	Course Structure	Course Outcomes
	elective's impact on their well-being through a post-course essay.	▪ Qualitative themes discussed in students' post-course essays reflected these effects. Themes included: reconnection between mind and body, community in a competitive environment, increased mindfulness, confidence in the use of mind-body skills with patients, and stress management.

Supplemental Table 9: Reproductive Health Medical Student Electives

Study	Course Structure	Course Outcomes
Meites, 2002<br>A student-initiated interactive course as a model for teaching reproductive health.	▪ Nine-week long course with lectures on reproductive health.<br>▪ Purpose of the course was to teach core competences in reproductive health, several of which parallel competencies identified by the Association of Professors of Gynecology and Obstetrics (APGO).<br>▪ Students presented various topics on reproductive health at a reproductive health fair at the end of the course	▪ Students completed a yes/no survey following completion of the course.<br>▪ The majority of students self-reported increases in comfort discussing reproductive issues such as contraception and sexual orientation.
Caro-Bruce, 2006<br>Addressing gaps in abortion education: a sexual health elective created by medical students.	▪ Consisted of 10 lectures.<br>▪ Speakers included medical school faculty and outside experts, including clergy members and a counselor from a clinic that provides abortions.<br>▪ Students were also required to attend a half-day shadowing experience at a local abortion clinic.	▪ Of the 50 students who enrolled in the course, 29 (58%) responded to the course survey. A five point Likert scale was used to assess the course (1= excellent, 5=inadequate/poor). Students gave the course an overall rating of 1.8 and a 1.9 on the "elective enhanced my understanding of the topics."<br>▪ The lectures on "First trimester surgical abortion" and "Voluntary HIV counseling and testing" received the highest rating of all the lectures at 1.6.

Supplemental Table 10: Miscellaneous Medical Student Electives

Study	Course Structure	Course Outcomes
Crandall, 2003 Applying theory to the design of cultural competency training for medical students: a case study	▪ One-year long pilot elective course titled "Culture and Diversity" for second-year students.<br>▪ Consisted of 20 two- to three–hour sessions during the year incorporating interactive lectures, videos, simulation, demonstration, role-plays, workshops, community-based service–learning, and online problem-based learning cases.<br>▪ Guests included local, national, and international experts on cultural influences on health.<br>▪ Assignments included a critical and reflective journal entry for each session, an interview with an individual culturally different from the student and reflection essay, observed interviews using interpreters, and a self-directed service-learning project.	▪ Students were asked to complete questionnaires during the first and last sessions of the course.<br>▪ Students reported statistically significant improved knowledge, attitudes, and skills related to cultural competence.
Quraishi, 2005 The Health Policy and Legislative Awareness Initiative at the Pennsylvania State University College of Medicine: theory meets practice.	▪ Semester-long course for 1st and 2nd year medical students<br>▪ Featured local and national health policy experts that delivered lectures on various topics and engaged in discussions around assigned readings<br>▪ Over the course of a semester, all participating students also complete a legislative internship in the office of a state legislator<br>▪ All students are required to either work with their legislative office on a bill that is currently being drafted or submit a policy resolution to a national medical organization as a final course requirement	▪ Students completed surveys on the first day of the course, on the last day of the course, and one year after completing the course.<br>▪ Students reported that they read health policy literature more often, were more interested in pursuing a policy dual degree (i.e. MPH), and had a better understanding of how legislation affects the practice of medicine.
Mouradian, 2006 A new oral health elective for medical students at the	▪ Lectures were given by medical school and dental school faculty to increase medical student knowledge of issues in oral health<br>▪ Each elective session was followed by a clinical laboratory experience	▪ The course received positive feedback, with a mean overall student rating of 4.67 out of 5<br>▪ Student confidence in identifying oral disease was significantly better after

Study	Course Structure	Course Outcomes
University of Washington.	or a patient interview exercise	completion of the course ($p<0.01$)
Sheu, 2010<br>A model for interprofessional health disparities education: student-led curriculum on chronic hepatitis B infection.	▪ Consisted of a didactic elective, clinical skills elective, and clinical practicum targeted towards pre-clinical medical, pharmacy, nursing, and dental students.<br>▪ The didactics portion included 10 one-hour sessions covering topics such as HBV epidemiology, transmission, prevention and treatment. Additional topics included cultural competency and community advocacy.<br>▪ The clinical skills sessions then culminated in a clinical practicum in which students attended outreach clinics to screen and vaccinate vulnerable patients from the Asian/Pacific Islander population.	▪ Of the 477 students who participated in the elective between 2004 and 2009, 24% were medical students.<br>▪ Of the 477 students, 78% participated in the clinical practicum. Between October 2008 and May 2009, students participated in an average of 1.89 clinics (SD ±1.32).<br>▪ Medical students volunteered twice as often as students from other schools.<br>▪ Between November 2007 to May 2009, students educated and screened a total of 804 patients.
Dussán, 2009<br>Effects of a refugee elective on medical student perceptions	▪ Community physicians, social agency workers, and other local experts in the field of refugee advocacy and care delivered lectures to medical students.<br>▪ Students also had contact with refugees in the classroom setting through a refugee discussion panel with local area refugees.	▪ All medical students filled out a survey pertaining to their attitudes and beliefs regarding refugee health. A 5-point Likert scale from strongly agree to strongly disagree was used.<br>▪ Participants in the elective were significantly more likely to feel comfortable interacting with foreign-born patients and identify cultural differences in understanding medical/mental health conditions than non-participants.
Pearson, 2009<br>Teaching the Art of Healing	▪ Five sessions over eight weeks for 2nd year medical students<br>▪ Suggested readings, in-class exercises, and small group discussions led by a physician are used for each session<br>▪ Course teaches the importance of listening, dealing with grief and loss, and finding fulfillment in their work	▪ No formal evaluation of the course<br>▪ Students commented that the course rekindled their reasons for going into medicine, including serving others and taking the time to understanding their patients' concerns
Robinson, 2010	▪ A month-long elective targeted to 1st	▪ Pre and post-tests were

Study	Course Structure	Course Outcomes
Air Medical Transport Curriculum Provides Education for Medical Students.	and 2nd year medical students.<br>▪ Included three 6-hour fly-along sessions, 2 hours of lectures, 2 hours of case review, an 80-page syllabus listing articles and position papers, and a 3-page writing assignment.	conducted to measure increases in knowledge. The mean number of correct responses on the post-test was improved ($p < .001$).<br>▪ A five point Likert scale was used to assess student satisfaction with the course. All students were either satisfied (56%) or very satisfied (44%) with the lectures and case review.<br>▪ Nine students (75%) were very satisfied overall and 3 (25%) were satisfied.
Crump, 2010 Outcomes of a preclinical rural medicine elective at an urban medical school.	▪ Included once-a-month 2.5 hour evening sessions targeted towards 2nd year medical students.<br>▪ During each session, students gave presentations on pre-selected topics pertaining to rural medicine and a different visiting professor (all practicing rural physicians) gave commentary on the students' presentations.	▪ A 21 question survey to assess student perceptions and a 44-question true-false exam to test student knowledge was used before and after the elective.<br>▪ Students showed statistically significant increases in knowledge about rural practice.<br>▪ Of the 65 participants between 2005 and 2009, 25 (39%) chose family medicine as a specialty compared to 85 (7.7%) of the 1,103 medical student population overall.
Knox, 2013 Educating medical students about adolescent maltreatment.	▪ A nine-month elective course targeted to 1st year medical students<br>▪ Addressed the prevention, identification, reporting, and response to all forms of child and adolescent maltreatment.<br>▪ Psychologists, physicians, law enforcement, and child protective services (CPS) workers gave lectures during the course.	▪ A survey was administered at the end of the course to both participants in the course and non-participants. The survey used a 5-point Likert scale ranging from "Very Unprepared" to "Very Prepared."<br>▪ Students' actual ability to identify and report maltreatment was measured at both pre-test and post-test using analog vignettes, which showed significant improvements

Study	Course Structure	Course Outcomes
Vinci, 2013<br>The Quality and Safety Track: Training Future Physician Leaders.	▪ 10 sessions on quality improvement topics such as choosing measures, designing interventions, patient safety, and pay for performance.<br>▪ Students also work in groups of 4 to 6 students to complete a data analysis and literature review project using publicly available data on a quality improvement topic of their choice.	▪ The Quality Improvement Knowledge Assessment Tool (QIKAT) of self-assessed comfort level with QI skills was completed before and after the elective.<br>▪ Students showed statistically significant increases in their self-perceived ability to make changes in a system, use small cycles of change, and to implement a Plan-Do-Study-Act cycle.
Timm, 2014<br>Book discussion course: timely topics for medical students.	▪ Three sessions featuring discussions led by medical school librarians<br>▪ Students were asked to read several case studies, stories, and vignettes on topics such as medical realities of terminal illness, complex family dynamics, rural medicine, and ethical dilemmas	▪ The eight students in the course were given a survey consisting of eight Likert scale questions and several open ended questions<br>▪ Student’s strongly agreed that the course made them think about doctor-patient issues and fostered active participation during class time
